# Wnt/Calcium Signaling Mediates Axon Growth and Guidance in the Developing Corpus Callosum

**DOI:** 10.1002/dneu.20846

**Published:** 2010-10-08

**Authors:** B Ian Hutchins, Li Li, Katherine Kalil

**Affiliations:** 1 Neuroscience Training Program, University of Wisconsin-MadisonMadison, Wisconsin; 2 Department of Anatomy, University of Wisconsin-MadisonMadison, Wisconsin

**Keywords:** corpus callosum, calcium signaling, Wnt, CaMKII, growth cone

## Abstract

It has been shown *in vivo* that Wnt5a gradients surround the corpus callosum and guide callosal axons after the midline (postcrossing) by Wnt5a-induced repulsion via Ryk receptors. In dissociated cortical cultures we showed that Wnt5a simultaneously promotes axon outgrowth and repulsion by calcium signaling. Here to test the role of Wnt5a/calcium signaling in a complex *in vivo* environment we used sensorimotor cortical slices containing the developing corpus callosum. Plasmids encoding the cytoplasmic marker DsRed and the genetically encoded calcium indicator GCaMP2 were electroporated into one cortical hemisphere. Postcrossing callosal axons grew 50% faster than pre-crossing axons and higher frequencies of calcium transients in axons and growth cones correlated well with outgrowth. Application of pharmacological inhibitors to the slices showed that signaling pathways involving calcium release through IP3 receptors and calcium entry through TRP channels regulate post-crossing axon outgrowth and guidance. Co-electroporation of Ryk siRNA and DsRed revealed that knock down of the Ryk receptor reduced outgrowth rates of postcrossing but not precrossing axons by 50% and caused axon misrouting. Guidance errors in axons with Ryk knockdown resulted from reduced calcium activity. In the corpus callosum CaMKII inhibition reduced the outgrowth rate of postcrossing (but not precrossing) axons and caused severe guidance errors which resulted from reduced CaMKII-dependent repulsion downstream of Wnt/calcium. We show for the first time that Wnt/Ryk calcium signaling mechanisms regulating axon outgrowth and repulsion in cortical cultures are also essential for the proper growth and guidance of postcrossing callosal axons which involve axon repulsion through CaMKII. © 2010 Wiley Periodicals, Inc. Develop Neurobiol 71: 269–283, 2011.

## INTRODUCTION

Wnt proteins are a family of morphogens that have recently been shown to function as axon guidance cues (Salinas and Zou,^2008^). Wnt5a, through the Ryk receptor, mediates the guidance of efferent corticospinal and callosal axons (Liu et al.,^2005^; Keeble et al.,^2006^; Zou and Lyuksyutova,^2007^). Knockout of the Ryk receptor causes misrouting of corpus callosal axons *in vivo* after axons have crossed the midline (Keeble et al.,^2006^). Gradients of Wnt5a surround the callosum and corticospinal tract and Wnt5a repels cortical axons in explant cultures. Thus in the callosum of knockout mice lacking Ryk receptors guidance errors were attributed to disruption of Wnt5a/Ryk-mediated axon repulsion. However, the signaling mechanisms downstream of Ryk in the context of axon growth and guidance were completely unknown (Liu et al.,^2005^; Keeble et al.,^2006^). Recently we found that Wnt5a gradients not only repel cortical axons in an *in vitro* turning assay but at the same time increase their rates of outgrowth (Li et al.,^2009^), consistent with the propulsive model of Wnt5a signaling (Zou and Lyuksyutova,^2007^). Further, we found that Ryk receptors are essential for the growth promoting and repulsive guidance effects of Wnt5a gradients and that these effects are mediated by calcium signaling pathways.

We considered it important to test the *in vivo* relevance of the Wnt/calcium signaling mechanisms that we previously identified in dissociated cortical cultures (Li et al.,^2009^). In dissociated cultures neurons are maintained in a simplified environment and effects of molecular cues on axons are tested one at a time. *In vivo*, axons encountering a complex environment must respond to a multitude of signals. Thus responses of axons in culture may not reflect how they behave in a complex neural pathway *in vivo* (Gomez and Zheng,^2006^). For example, knocking down calcium/calmodulin-dependent protein kinase I (CaMKI) in dissociated cultures decreases axon elongation (Ageta-Ishihara et al.,^2009^; Davare et al.,^2009^; Neal et al.,^2010^). In contrast, knocking down CaMKI *in vivo* decreases callosal axon branching into cortex without affecting rates of axon elongation (Ageta-Ishihara et al.,^2009^). We therefore used developing cortical slices that contained the entire callosal pathway through the sensorimotor cortex, which permitted imaging of intact callosal axons extending along their entire trajectory (Halloran and Kalil,^1994^). Another important advantage of the slice preparation is that experimental manipulations of molecular signaling pathways can be carried out at specific locations and at specific times in development. In the present study we identified Wnt/calcium signaling mechanisms that mediate growth and guidance of callosal axons.

## MATERIALS AND METHODS

### Slice Preparation and Electroporation

Cortical slice injection and electroporation methods were adapted from (Uesaka et al.,^2005^). Briefly, slices were obtained from P0 hamster brains. Pups were anesthetized on ice and the brains are rapidly removed into ice-cold Hank's Balanced Salt Solution (HBSS, Invitrogen). The brains were encased in 4% agar and solidified on ice. Coronal slices (400 μm) through the forebrain are cut on a vibratome and collected in cold HBSS (Halloran and Kalil,^1994^). Slices were then cultured on 0.4 μM membrane inserts (Millipore) in plating medium containing 5% fetal bovine serum (Invitrogen), 2% B27 supplement (Invitrogen), and 1% liquid glutamine-penicillin-streptomycin (Invitrogen) in Neurobasal medium (Invitrogen) and were maintained at 37°C at 5% CO_2_. After recovering for up to 1 day *in vitro*, slices containing the corpus callosum were placed into the well of an open chamber fitted with a platinum electrode bottom (CUY700P10E, Nepagene). Plasmids (1 μg μL^−1^) encoding DsRed2, a cytoplasmic fluorescent protein, were pressure injected (from a glass pipette with a 25 μm tip for 20 ms at 12 PSI) alone into several sites within a single cortical hemisphere or were coinjected with Ryk siRNA (diluted to 5 μg μL^−1^) to knock down Ryk receptors. Alternatively, plasmids encoding GCaMP2 (Addgene plasmid 18927) or EGFP-CaMKIIN were used to visualize calcium activity or inhibit CaMKII, respectively. For ratiometric imaging experiments, DsRed2 and GCaMP2 were coinjected into slices with or without Ryk siRNA. About 88% of axons expressing GCaMP2 also expressed DsRed2, indicating a high cotransfection efficiency. Electroporation was carried out with a square wave pulse generator (CUY-21, Nepagene) which delivered 20 pulses of 10-ms duration at 4 Hz and 50 V. Slices were then allowed to recover for 48 h before imaging. At P2 efferent cortical axons are extending toward and into the corpus callosum but have not projected across the midline. Thus examination of axons 48 h after electroporation allowed us to follow callosal axons across the midline and contralaterally.

### Experimental Reagents

Stock solutions were prepared by dissolving drugs in water or dimethyl sulfoxide (DMSO) according to the recommendations of the manufacturer. Stock solutions were then diluted into ACSF (described below) and perfused over slice cultures. The following reagents were used: 2-aminoethoxydiphenyl borate (2-APB, Calbiochem), SKF96365 (Alexis Biochemicals), bovine serum albumin (BSA, Sigma), recombinant protein Wnt5a (R&D systems), ON-TARGET*plus* SMARTpool mouse Ryk siRNA (Dharmacon), and a second, independent Ryk siRNA pool (Santa Cruz Biotechnology).

### Imaging of Callosal Axons

Slices were placed in an open perfusible chamber (Warner Instruments) and viewed either with an Olympus (Center Valley) Fluoview 500 laser-confocal system mounted on an AX-70 upright microscope with a 40× plan fluor water immersion objective (outgrowth and calcium imaging experiments) or a Nikon TE300 inverted microscope with a 20× objective (outgrowth experiments only). Temperature was maintained at 37°C with a temperature controller (Warner Instruments). A perfusion system was used for continuous oxygenation of the heated artificial cerebrospinal fluid (ACSF, containing 124 m*M* NaCl, 24 m*M* NaHCO_3_, 3 m*M* KCl, 1.25 m*M* NaH_2_PO_4_, 2 m*M* CaCl_2_, 1.5 m*M* MgCl_2_, 10 m*M* glucose, and 20 m*M* HEPES) to which pharmacological reagents (2-APB, 50 μ*M*; SKF96365, 3 μ*M*) were added. Perfusion of the slices with medium was carried out at a flow rate of 2 mL min^−1^. Time lapse images were obtained every 5–15 s for measurements of axon outgrowth for up to 90 min. For calcium imaging, images were obtained twice a second on the Fluoview 500 system during free-scan mode for up to 30 min. Long term (3 h) treatments with 2-APB or SKF96365 were returned to the incubator and imaged at the beginning and end of this treatment to assess effects on axon trajectories.

### Measurements of Calcium Activity

Calcium activity was measured as the average fluorescence pixel intensity (F) in an axon region divided by the baseline fluorescence in that region (F0). Background fluorescence was measured frame-by-frame and was subtracted from measurements of fluorescence intensity. To minimize the effects of any morphological changes that could affect fluorescence measurements through changes in volume, the baseline (F0) was calculated as a shifting average of the fluorescence intensity over a 30-frame window.

To choose a threshold that defined a calcium transient, we first simulated the number of false positive readings we would measure in a signal that was derived from Gaussian noise with a similar mean and standard deviation as our measured calcium signals. The number of false positive readings measured from our simulation of 50 calcium imaging experiments was acceptably low at a threshold of 3.5 standard deviations above baseline (corresponding to 1.8 false positive transients h^−1^). Thus, calcium transients were defined as fluorescence signals (F/F0) that exceed 3.5 standard deviations above baseline, which were confirmed by frame-by-frame analysis of the time-lapse images. For ratiometric experiments, slices were co-electroporated with DsRed2 and GCaMP2. Fluorescence images of DsRed2 acquired simultaneously with each frame of GCaMP2 fluorescence. Ratiometric measurements (*R*) were obtained by dividing the GCaMP2 fluorescence value by the fluorescence value of DsRed2. Frame-by-frame background subtraction was performed for each indicator as described above. Calcium signals (*R*/*R*0) were then measured as the percent change from a shifting average baseline (*R*0) of the ratiometric measurements as described above for nonratiometric measurements. Although expression levels of GCaMP2 varied from cell to cell, this did not affect the frequency of calcium transients reported. Raw baseline fluorescence did not correlate with frequency (Spearman correlation coefficient *r* = 0.09, *p* = 0.69). We validated our calcium transient measurements with additional power spectral density analysis (Uhlen,^2004^; Bortone and Polleux,^2009^), which measures periodicity in a time series signal without an arbitrary definition of a transient. This analysis (our unpublished observations) confirmed higher periodicity as measured by average relative power in calcium signals in contralateral vs. ipsilateral axons at a frequency of 15 per hour, the frequency of calcium transients evoked by Wnt5a *in vitro* (Li et al.,^2009^).

### Quantification of Axon Outgrowth and Trajectories

**Figure 3:**
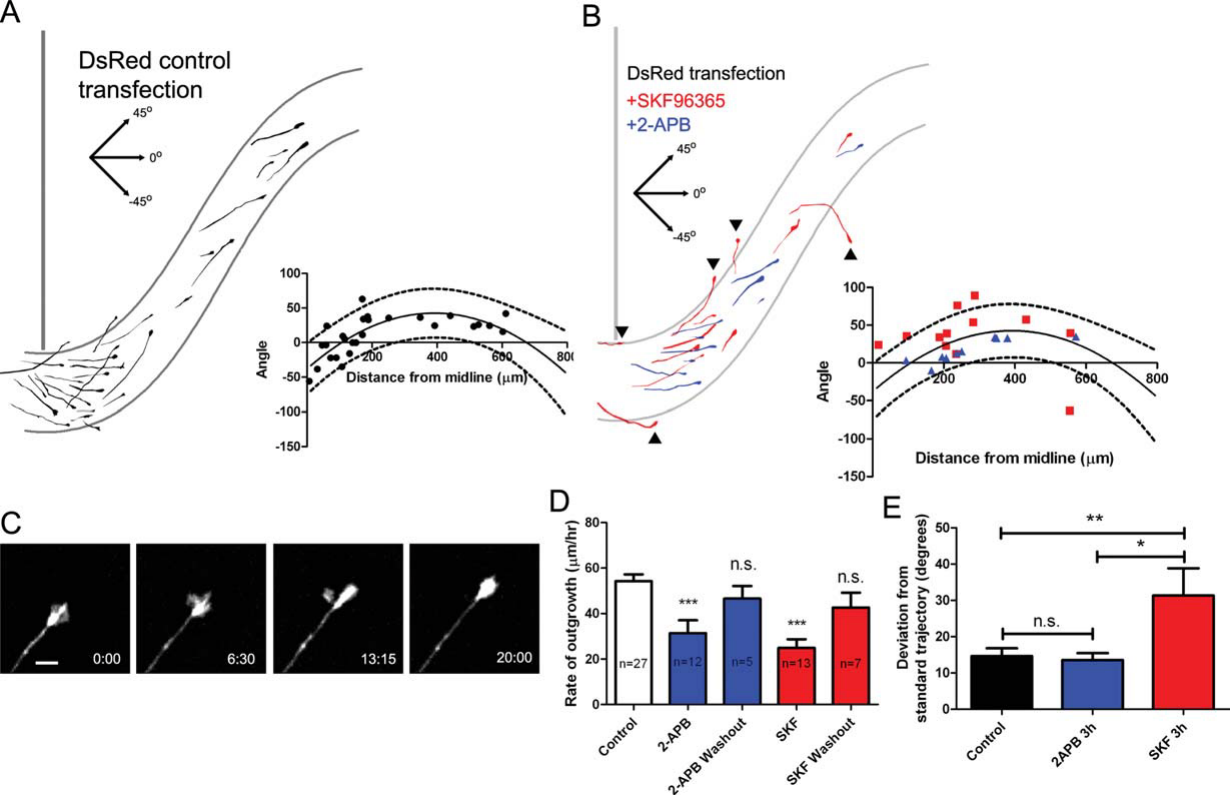
Blocking IP3 receptors and TRP channels reduces rates of postcrossing axon outgrowth and blocking TRP channels leads to axon guidance defects. (A) Tracings of cortical axons expressing DsRed2 in the contralateral corpus callosum. Axons from different experiments were traced and overlaid on a single outline of the corpus callosum. Curved lines, border of the corpus callosum; vertical line, midline. (A, inset) Plot of growth cone distance from the midline versus axon trajectory (see methods) in control experiments. The solid line represents a quadratic regression curve which describes the standard trajectory taken by axons in control experiments; the dashed lines represent the 90% prediction interval of the regression curve. (B) Tracings of cortical axons in slices treated with 2-APB (blue) conformed to the standard trajectory of callosal axons without deviating significantly (see Methods) while axons in slices treated with SKF96365 (red) deviated dorsally toward the induseum griseum or ventrally toward the septum or lateral ventricle or cortical plate in many cases (5 of 12 axons, arrowheads). (B, inset) Plot of growth cone distance from the midline versus axon trajectory in axons in slices treated with SKF96365 (red) or 2-APB (blue). The solid line indicates the standard trajectory derived from control axons and the dashed lines are the 90% prediction interval. (C) Time lapse images of a growth cone expressing DSRed2 extending through the callosum after crossing the midline, during treatment with 2-APB. Scale bar, 10 μm. (D) Rates of outgrowth of callosal axons under control conditions, during bath application of 2-APB or SKF96365, or after washout. *n* = number of axons. (E) Measurement of the average deviation of axons treated with 2-APB (*n* = 10), SKF96365 (*n* = 12) or medium (control, *n* = 27) from the standard trajectory. ***p < 0.001, One way ANOVA with Dunnett's posttest. **p < 0.01, *p < 0.05 One way ANOVA with Newman-Kewls posttest.

**Figure 4:**
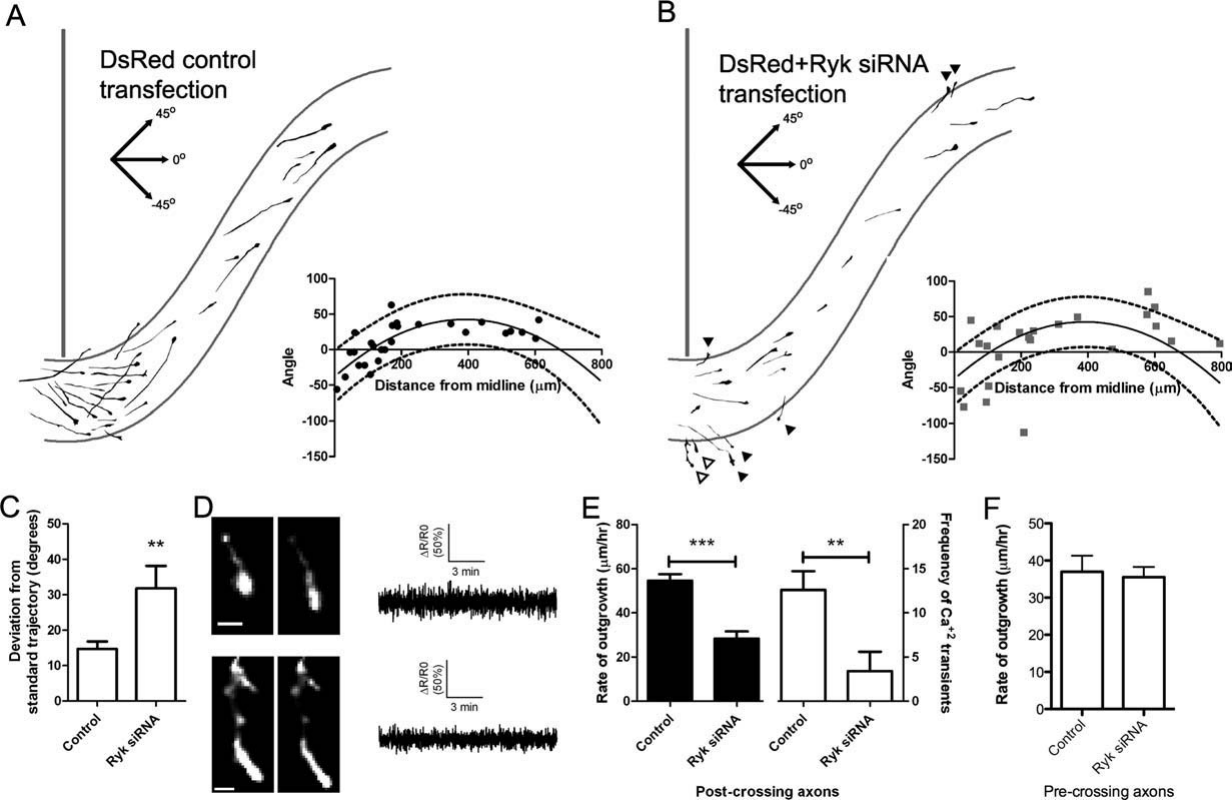
Ryk knockdown reduces frequencies of calcium transients, slows rates of axon extension, and causes axon guidance defects in post-crossing callosal axons. (A) Tracings of control cortical axons expressing DsRed2 [also shown in Fig. 3(A)] in the contralateral corpus callosum. (A, inset) Plot of growth cone distance from the midline versus axon trajectory in control experiments. The solid line represents a quadratic regression curve which describes the standard trajectory taken by axons in control experiments; the dashed lines represent the 90% prediction interval of the regression curve. (B) Tracings of cortical axons in slices electroporated with DsRed2 and anti-Ryk siRNA. Many of these axons with Ryk expression knocked down deviated dorsally toward the induseum griseum or cortical plate or ventrally toward the septum (arrowheads; anti-Ryk siRNA: 7 of 23 axons). (B, inset) Plot of growth cone distance from the midline versus axon trajectory in Ryk knockdown experiments. The solid line indicates the standard trajectory derived from control axons and the dashed lines are the 90% prediction interval. (C) Measurement of the average deviation of axons expressing with DSRed2 plus anti-Ryk siRNA (*n* = 23) or DsRed2 alone (control, *n* = 27) from the standard axon trajectory. (D, left) Growth cones electroporated with Ryk siRNA, also co-expressing DsRed2 (shown in left panels) and GCaMP2 that are extending toward the septum (shown in (B) with hollow arrowheads). Scale bars, 10 μm. (D, right) Tracings of calcium signals measured by ratiometric imaging showing that neither of these neurons express calcium transients. (E) Quantifications of rates of axon outgrowth (left, black; *n* = 27 for controls and 22 for Ryk siRNA experiments) and frequencies of calcium transients (right, white; *n* = 14 for controls and 10 for Ryk siRNA experiments) in post-crossing callosal axons. Units are transients h^−1^. (F) Quantification of precrossing axon outgrowth in slices electroporated with DsRed or DsRed plus Ryk siRNA (*n* ≥ 6 axons from at least two slices). ***p < 0.001, **p < 0.01, *t* test.

**Figure 5:**
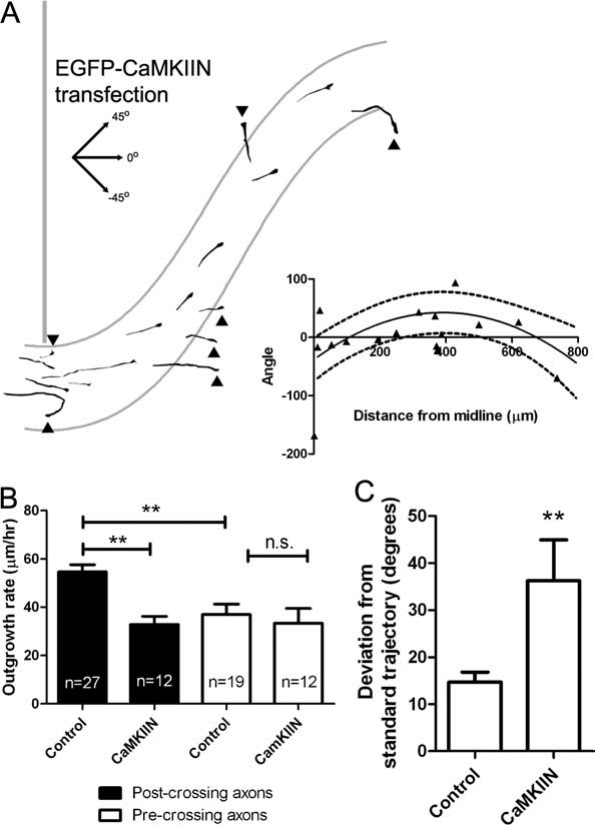
CaMKII regulates cortical axon outgrowth and guidance in the corpus callosum. (A) Tracings of cortical axons in slices electroporated with EGFP-CaMKIIN, which deviated dorsally toward the induseum griseum or cortical plate or ventrally toward the lateral ventricle in many cases (arrowheads; 7 of 16 axons). (A, inset) Plot of growth cone distance from the midline versus axon trajectory in axons in slices electroporated with EGFP-CaMKIIN.The solid line indicates the standard trajectory derived from control axons and the dashed lines are the 90% prediction interval. (B) Rates of axon outgrowth in cortical neurons expressing DSRed2 (control) or EGFP-CaMKIIN in pre- or postcrossing callosal axons. *n* = number of axons. **p < 0.01, One way ANOVA with Bonferroni's posttest. (C) Measurement of the average deviation of axons expressing with EGFP-CaMKIIN (*n* = 16) or DsRed2 (control, *n* = 27) from the standard trajectory. **p < 0.01, t test.

Outgrowth was measured as the displacement in μm of the distal tip of the growth cone between the first and last frames of an imaging session divided by the duration of that session. Overexpression of multiple constructs (DsRed and GCaMP2) had no deleterious effect on rates of postcrossing axon outgrowth, which grew at 114% of the rate of controls expressing only one construct (a nonsignificant increase). Trajectories were measured as the angle between the horizontal axis of the slice and the distal 20 μm of callosal axons, plotted versus the horizontal distance from the midline. These data were best fit by a quadratic regression curve which we used to describe the standard trajectory taken by control axons in our control experiments. Deviation away from the standard trajectory of control axons was measured as the difference in degrees between the measured angle of an axon and the angle predicted by the regression curve for an axon at that distance from the midline. Plots of the trajectories of axons from this study are shown in Figures 3–5 alongside the best-fit regression curve and 90% prediction intervals describing the trajectories of control axons. Individual axons in our experimental manipulation groups were considered to be significantly deviating from the standard trajectory if they fell outside the 90% prediction intervals [Fig. 3(A)]. These axons are shown as deviating from the corpus callosum in our tracings (Figs. 3–5) and are marked with arrowheads. Unless otherwise noted, n is the number of axons from at least three independent experiments.

### Dissociated Cortical Neuron Cultures and Wnt5a Experiments

Culture of dissociated cortical neurons and bath application experiments with Wnt5a were performed as previously described (Li et al.,^2009^). Briefly, cortical neurons were dissociated from P0 hamster sensorimotor cortex and electroporated with EGFP-CaMKIIN plasmids with an Amaxa Nucleofector. These neurons were plated onto coverslips coated with 0.5 mg mL^−1^ poly-d-lysine (Sigma) and 20 μg mL^−1^ laminin (Sigma/Invitrogen) at a density of 2000–7000 per cm^2^ and were incubated in 5% CO_2_ and 9% O_2_ at 37°C for 2 days. For long term axon outgrowth assays, 400 ng mL^−1^ Wnt5a in 0.5% BSA is PBS, or BSA alone, was then added to the cultures. Cultures were then incubated for 72 h before fixation. Axon lengths were measured in neurons expressing EGFP-CaMKIIN or in untransfected neurons from the same dish as a control.

### Dunn Chamber Axon Guidance Assay and Analysis

For Dunn chamber axon guidance assays, P0 hamster cortical neurons were grown on appropriately coated (see above) 22-mm^2^ No. 1.5 coverslips (Corning) at a low density (10 k cells/well in a six well plate (Falcon). Assembly of the Dunn chamber (Hawksley, UK) was modified from previous studies (Yam et al.,^2009^). Dunn chambers were rinsed by serum-free medium once and then both inner and outer wells were filled by serum-free medium. To secure coverslips with neurons on the chamber, silicon sealant (Dow Corning) was applied at ∼0.5 cm from the border of outer well but omitted at one side to form a slit later for draining and refilling the outer well. A coverslip with neurons was inverted over the Dunn chamber leaving a narrow slit at the edge without the sealant. Media at the outer well was aspirated and then medium with 400 ng mL^−1^ Wnt5a was added to the outer well. The narrow slit was sealed by fixing a small piece of parafilm (American National Can) to the chamber with sealant. Images were acquired immediately after Dunn chamber assembly and 2 h later with a 20 × 0.5 numerical aperture (NA) Plan Fluor objective mounted on a Nikon TE300 Quantum inverted microscope equipped with a Photometrics Cascade II: 512. All dissociated neurons in the bridge region of the Dunn chamber were imaged in each experiment. Dunn chambers were kept at 37°C during imaging.

We followed similar criteria as (Yam et al.,^2009^) for data analysis. Briefly, we only included individual axons with >10 μm straight distal end and with net outgrowth >5 μm h^−1^. The outgrowth of axons was measured by tracing axons from the original position of growth cones to their final position. The original direction of axon outgrowth was defined by the direction of the 10-μm distal axon segment. The initial angle (0°–180°) was defined by the angle between the original direction of axon outgrowth and the direction of the gradient. Final angles were calculated as the angles between the original direction of axon outgrowth and a line connecting the original position of the growth cone and its final position. When axons turned towards the gradient, the angle turned was assigned as positive and when axons turned away from the gradient, the angle turned was negative. Images presented in figures were rotated to so that the gradient increases along the *y*-axis to more easily compare responses of axons.

### Western Blotting

ON-TARGETplus nontargeting pool siRNA was purchased from Dharmacon. Neurons obtained from P0 hamsters were electroporated with 100 pmol control siRNAs and after 4DIV, proteins were extracted by RIPA buffer (Thermo Fisher Scientific) supplied with protease inhibitor cocktail tablets (Roche). Blots were blocked with 3% milk (Lab Scientific) and 3% BSA (Sigma) for 2 h and then incubated with mouse anti-human βIII tubulin (1:500, Millipor Bioscience Research Reagents) at 4°C overnight and goat anti-mouse-HRP (1:10,000, Jackson ImmunoResearch) for 1 h. ECL plus (GE health) was used to stain tubulin and Ryk receptors.

### Statistical Analysis and Image Processing

Graphs and statistical analysis were performed with Prism (GraphPad) statistical analysis software. Unless otherwise noted, comparisons between two groups were made with Student's *t* test and comparisons between multiple groups were made with a one-way ANOVA with Dunnett's posttest. Measurements are given in mean ± SEM unless otherwise noted. Images were modified with a low-pass filter in MetaMorph to reduce single-pixel noise. The images presented in figures were enhanced with brightness-contrast adjustments in Adobe (Mountain View, CA) Photoshop, and with Flatten Background and Sharpen adjustments in MetaMorph for slice images taken from the Nikon epifluorescence system [Fig. 3(C)].

## RESULTS

### Calcium Signaling Regulates Growth and Guidance of Callosal Axons

**Figure 1:**
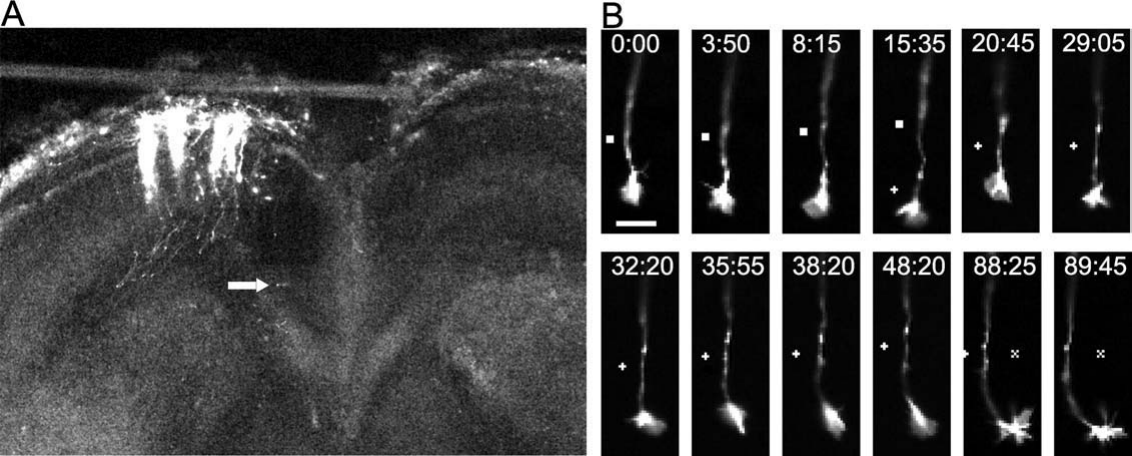
Visualization of individual callosal axons and their growth cones as they extend through the callosum. (A) A low power confocal image of a cortical slice at 3DIV, after electroporation of cortical neurons with DsRed2 performed on the slice from a P0 hamster. Note that individual efferent axons can be clearly visualized. Arrow indicates location of the cortical growth cone imaged at higher power in the time lapse sequence in (B). (B) Turning behaviors in images at bottom are clearly visible as are filopodia and lammellipodia. Scale bar, 10 μm. ▪, +, X, reference points.

As a first step we labeled small numbers of cortical neurons by electroporation with plasmids encoding the cytoplasmic marker DsRed2 into one hemisphere of cortical slices from P0 hamster brains and used live imaging to follow the extension of individual axonal growth cones in the corpus callosum. As shown in Figure 1(A), electroporation labeled a small region of the sensorimotor cortex including Layers 2–3 and 5 that give rise to subcortical, horizontal, and callosal axons. Time lapse imaging showed that callosal growth cones (*n* = 46) exhibit continual motility, forward advance, and turning behaviors (Supporting Information, Movie 1). In sequences [Fig. 1(B)] lasting up to 1.5 h we measured rates of growth cone advance in different regions of the callosum. Growth cones that have not yet crossed the midline (precrossing) consistently advance more slowly (36.9 ± 4.3 μm h^−1^, *n* = 19) than those that have crossed the midline (post-crossing) (54.6 ± 2.9 μm h^−1^, *n* = 27) Labeled axons followed the trajectory of the callosal pathway as a whole [Fig. 3(A)].

**Figure 2:**
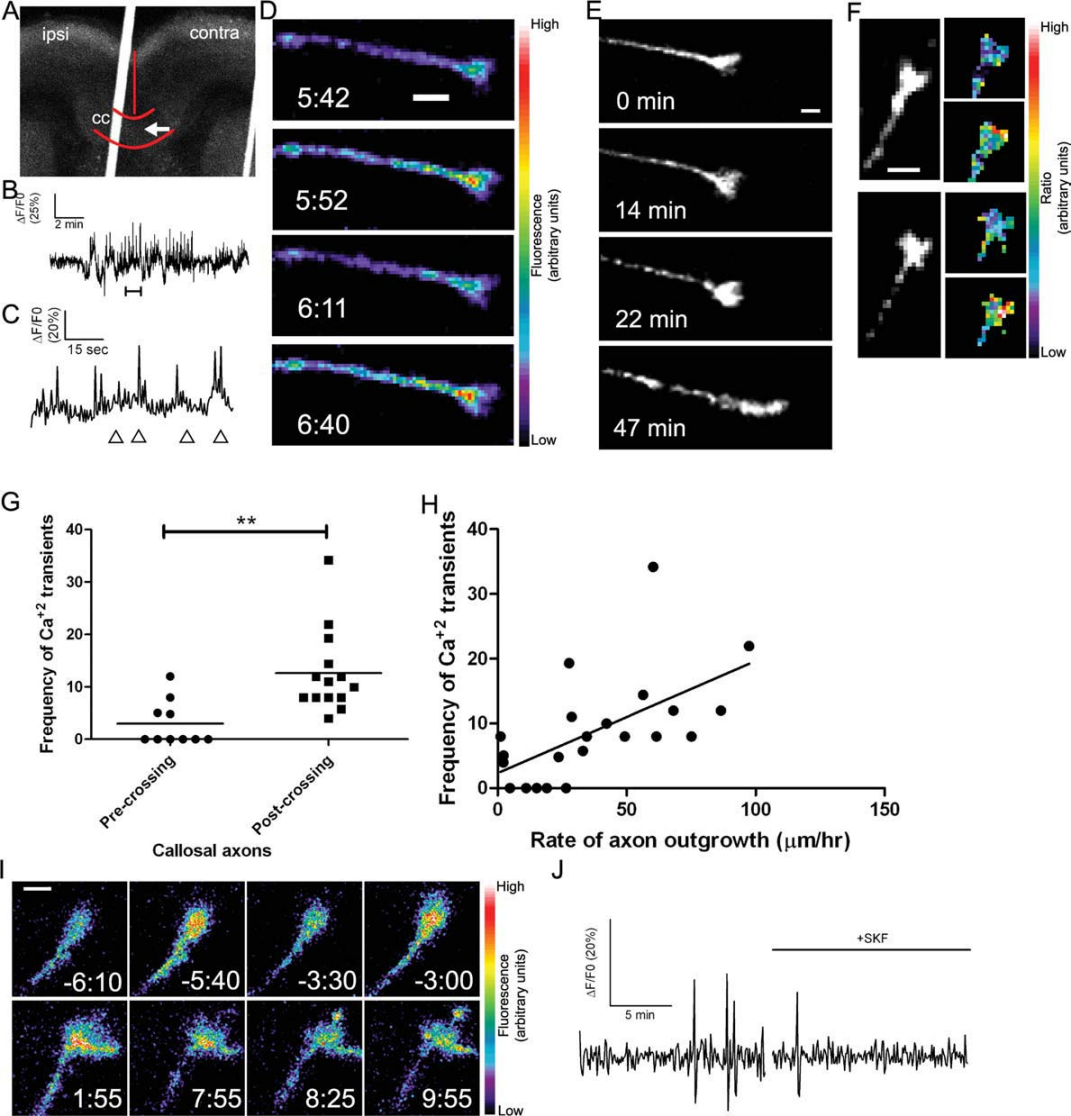
Callosal axons express spontaneous calcium transients that are correlated with rates of axon outgrowth. (A) A coronal cortical slice in which plasmids encoding GCaMP2 were injected and electroporated into the left cortex (ipsi). The arrow indicates the position of the growth cone imaged in B–D, which had crossed the midline. Red curves indicate the borders of the corpus callosum (cc) and the midline. The white line is autofluorescence from the slice holder used in live cell imaging. (B) Tracing of calcium activity measured by the change in GCaMP2 fluorescence over baseline. Calcium activity increases after a few minutes of imaging. (C) Tracing of calcium activity from (B) zoomed in to the time period indicated by the bracket (B, bottom). (D) Fluorescence images of the growth cone from (B–C) at the time points indicated by arrowheads in (C). (E) Within 20 min of the onset of calcium activity shown in (B) the axon begins to rapidly advance through the contralateral callosum. (F) Examples of single calcium transients measured by ratiometric imaging in growth cones coexpressing DsRed2 and GCaMP2. (G) Plot of frequencies of calcium transients in pre-crossing or post-crossing callosal axons. **p < 0.01, *t* test. All frequencies in units of transients h^−1^. (H) Scatter plot of the frequency of calcium transients versus the rate of axon outgrowth in individual callosal axons. The line represents the least-squares linear regression (slope significantly non-zero, *p* < 0.01). (I) An example of spontaneous calcium transients (top row) which are attenuated by application of SKF (time 0:00, bottom rows). (J) Tracing of calcium activity in the growth cone shown in (I) before and after application of SKF. Scale bars, 10 μm except I, which is 5 μm. Pseudocolor calibration bars indicate fluorescence intensity (D) or ratio of GCaMP2 to DsRed2 fluorescence intensities (F) in arbitrary units.

We next asked whether growth cones extending in the corpus callosum express spontaneous calcium activity as observed in dissociated cortical cultures (Tang et al.,^2003^; Tang and Kalil,^2005^; Hutchins and Kalil,^2008^). Calcium activity was measured in post-crossing axons and growth cones by electroporating GCaMP2, a genetically encoded calcium indicator (Tallini et al.,^2006^; Mao et al.,^2008^), into one hemisphere of the sensorimotor cortex [Fig. 2(A)]. Growth cones showed continuous motility and forward advance while expressing repetitive calcium transients [Fig. 2(D,E)] averaging 8.6 ± 1.7 per hour, a frequency somewhat lower than spontaneous activity of cortical growth cones in dissociated culture (11.1 transients h^−1^), (Hutchins and Kalil,^2008^). In some cases transients were detected in the axon as well as the growth cone [Fig. 2(D), Supporting Information, Movie 2] but in other cases changes in calcium activity were confined to a localized region of the growth cone [Fig. 2(F)] suggesting the expression of both global and localized calcium activity such as we had previously observed (Hutchins and Kalil,^2008^; Hutchins,^2010^).

We then asked whether the frequencies of calcium transients in callosal growth cones were related to axon growth rates. Since we found that the callosal axons extended significantly more slowly before vs. after the midline, we measured the frequencies of calcium transients in callosal growth cones in these two locations. Since GCaMP2 has a lower signal-to-noise ratio than small molecule calcium indicators such as Fluo-4, we included in our counts of calcium transients only those events that exceeded 3.5 standard deviations above baseline (see Methods). We found that precrossing axons growing at an average rate of 36.9 ± 4.3 μm h^−1^ had an average frequency of 2.99 ± 1.36 transient h^−1^ whereas postcrossing axons with an average growth rate of 54.6 ± 2.9 μm h^−1^ had an average frequency of 12.6 ± 2.12 transients h^−1^ [Fig. 2(G)]. Thus higher frequencies of calcium transients are well correlated with higher rates of callosal axon outgrowth [Figure 2(H)]. Amplitudes and durations of calcium transients were unrelated to rates of growth, indicating that frequency-dependent mechanisms in particular could regulate rates of axon advance through the corpus callosum.

Calcium release from internal stores and entry through TRP channels are important sources of calcium for regulating axon growth and guidance in response to environmental cues (Li et al.,^2005^,^2009^; Shim et al.,^2005^). Previously in dissociated cortical cultures we found that calcium influx through TRP channels mediates axon outgrowth and repulsive growth cone turning evoked by Wnt5a while calcium release from stores through IP3 receptors mediates axon outgrowth but not turning. To determine whether these calcium signaling mechanisms regulate axon outgrowth and guidance in the developing corpus callosum, we bath-applied 2-APB which is known to block calcium release from stores through IP3 receptors (Li et al.,^2005^,^2009^) and SKF96365 which is known to block TRP channels (Li et al.,^2005^,^2009^; Shim et al.,^2005^). *In vivo* suppression of spontaneous electrical activity in callosal axons was shown to decrease rates of axon outgrowth on the postcrossing but not the precrossing side of the callosum (Wang et al.,^2007^). Therefore in manipulating calcium activity, we focused on axon growth and guidance of postcrossing axons. In slices electroporated with plasmids encoding DsRed2, individual postcrossing callosal axons and their growth cones were imaged for 20 min in the presence of pharmacological inhibitors (see Fig. 3). Treatment with 2-APB caused no overt defects in the morphology or motility of the growth cones [Figure 3(C)] but slowed the rate of axon outgrowth to 31 ± 5.6 μm h^−1^ (*n* = 12 axons in five slices) an almost 50% reduction of control growth rate [Fig. 3(D)]. However, trajectories of individual callosal axons were similar to those of untreated controls [Fig. 3(B,E)]. Importantly, a 30-min washout of the 2-ABP restored the rates of axon outgrowth. Treatment with SKF96365 (*n* = 13 axons in five slices) also reduced rates of axon outgrowth by about 50% (24.9 ± 3.8 μm h^−1^) which were restored close to control levels after washout. Remarkably blocking TRP channels with SKF96365 caused severe misrouting of individual callosal axons [5 of 12, Fig. 3(B,E)]. As shown in Figure 3(B), tracing of axon trajectories showed that some axons turned prematurely toward the cortical plate while others turned inappropriately toward the septum or the ventricle. In several cases [one example shown in Fig. 2(I,J) and Supporting Information, Movie 3] we were able to apply SKF to cortical slices after imaging calcium activity in a postcrossing axon. In each case application of SKF attenuated ongoing calcium transients. Postcrossing axons treated with SKF had a frequency of calcium transients similar to that of precrossing axons (2.99 ± 1.36 per hour, *n* = 10 for precrossing control axons vs. 3.2 ± 2.33 per hour, *n* = 5 for SKF-treated postcrossing axons). This provides direct evidence that in callosal axons the growth and guidance defects observed after pharmacological treatment with SKF were the result of decreased calcium activity.

To quantify the deviation from the standard trajectory of axons in the contralateral callosum, we first plotted the distance from the midline of DsRed expressing growth cones in control slices versus axon trajectory (the angle between the line formed by the distal 20 μm of the axon and the horizontal axis of the slice). These angles [Fig. 3(A), inset] increased as axons grew away from the midline reflecting the fact that axons turn dorsally after descending into the callosum and crossing the midline. We then fit these data with a nonlinear regression curve which describes the standard trajectory of these axons. This allowed us to compare the actual angle of an axon at a given distance from the midline versus the angle predicted by the regression curve. As shown in Figure 3, axons in control and 2-APB-treated slices deviated very little from the standard trajectory (14.7° ± 2.2° and 13.6° ± 2.3°, respectively) while axons in SKF treated slices deviated significantly more (31.4° ± 7.5°, *p* < 0.01, One way ANOVA with Newman-Kewls posttest).

### Ryk Knockdown Disrupts Post-Crossing Axonal Calcium Signaling, Rates of Growth and Trajectories

Taken together, results thus far demonstrate the requirement of calcium signaling mechanisms in callosal axon outgrowth and guidance but not the specific involvement of Wnt5a signaling. In dissociated cortical cultures (Li et al.,^2009^) we found that knockdown of the Ryk receptor to Wnt5a prevented increased rates of axon outgrowth and repulsive growth cone turning evoked by Wnt5a. *In vivo* Ryk knockout mice were found to have guidance errors in callosal axons but the use of fixed material prevented studies of signaling mechanisms downstream of Ryk (Keeble et al.,^2006^). We used electroporation of Ryk siRNA to knock down Ryk in a small number of cortical axons to analyze cell autonomous functions of Ryk in a wild type background; to visualize these neurons and their axons, we co-electroporated DsRed. We used two pools of Ryk siRNA that we have extensively characterized in hamster cortical neurons (Li et al.,^2009^). Measurements of growth rates of fluorescently labeled axons revealed that postcrossing axons slowed their growth rates to 28.4 ± 3.2 μm h^−1^, about half the normal growth rate for axons that have crossed the midline [Fig. 4(E)]. Ryk knockdown had no effect on precrossing growth rates [Fig. 4(F)] where Ryk is known to be inactive (Keeble et al.,^2006^), demonstrating that electroporation with Ryk siRNA does not reduce rates of outgrowth in general but rather selectively reduces rates of growth in the regions where Ryk is active. To further test for off target effects of siRNA we compared Ryk expression levels in cortical neurons electroporated with a control pool of siRNA vs. mock transfection. Ryk expression levels were the same in these two groups (Supporting Information Fig. S1), arguing against off target effects of electroporation with siRNA. To assess whether Ryk knockdown disrupted the guidance of callosal axons we compared the trajectories of DsRed-labeled axons in control slices with axons in slices electroporated with Ryk siRNA [Fig. 4(A–C)]. We found that Ryk knockdown caused severe guidance errors in about a third of axons (*n* = 7 out of 23) analyzed [Fig. 4(A,B)]. The variable effect on axon guidance in siRNA-treated axons could be due to uneven knockdown of the Ryk receptor among axons. However, we were unable to test this possibility due to the ubiquitous expression of Ryk in the cortex (Keeble et al.,^2006^), which makes the detection of Ryk expression on single axons against this background unfeasible. Similar results were obtained with a second, independent pool of Ryk siRNA (Supporting Information Fig. S1). As shown in the axon tracings guidance errors of postcrossing callosal axons involved premature dorsal turning toward the overlying cortex or inappropriate ventral turning toward the septum.

Results obtained in dissociated culture (Li et al.,^2009^) showed that knocking down Ryk reduced the proportion of neurons that expressed calcium transients in response to application of Wnt5a. Are the outgrowth and guidance defects in the callosum of cortical slices in which Ryk was knocked down due to interference with Wnt evoked calcium signaling? To address this question we coelectroporated GCaMP2 with Ryk siRNA to monitor calcium activity in callosal growth cones in which Ryk/Wnt signaling has been disrupted. In these co-electroporated neurons [Fig. 4(D,E)] frequencies of calcium transients were reduced to 3.4 ± 2.2 transients h^−1^ compared to 12.6 transients h^−1^ for controls, a similar reduction in frequency to that caused by treatment with SKF. Remarkably, in several cases we found that in growth cones projecting inappropriately toward the septum, calcium transients were undetectable [Fig. 4(D)]. Taken together these results suggest that axon growth and guidance errors caused by Ryk knockdown result from attenuated calcium activity in callosal growth cones.

### CaMKII Regulates Repulsive Axon Guidance

Since we found previously that CaMKII is also a component of the Wnt/calcium signaling pathway (Li et al.,^2009^), (Supporting Information Fig. S2), we asked whether inhibiting CaMKII activity would cause growth or guidance defects of callosal axons. We reduced the activity of CaMKII by transfection of plasmids encoding a specific CaMKII inhibitor protein, EGFP-CaMKIIN (Chang et al.,^1998^; Tang and Kalil,^2005^). For postcrossing but not precrossing axons this treatment slowed the growth of callosal axons and caused guidance errors similar to those observed after Ryk knockdown. As shown in Figure 5(A,C) some transfected axons showed dramatic misrouting in which axons looped backwards in the callosum, prematurely extended dorsally toward the cortical plate or grew abnormally toward the ventricle. In these experiments we compared rates of precrossing (*n* = 12 axons in four slices) vs. postcrossing (*n* = 12 axons in five slices) callosal axons [Fig. 5(B)] and found that rates of postcrossing axon outgrowth were reduced by about 50% (36.2 ± 4.0 vs. 54.6 ± 2.9 μm h^−1^ for control axons) but rates of precrossing axon outgrowth were unaffected [Fig. 5(B)].

**Figure 6:**
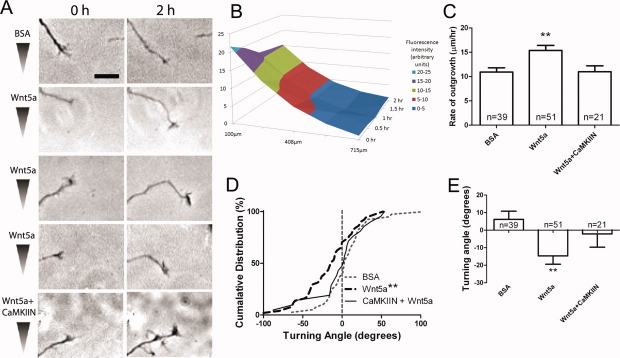
CaMKII activity is required for repulsive growth cone turning away from a gradient of Wnt5a. (A) At left, cortical growth cones responding to Wnt5a gradients in Dunn chambers over 2 h. Images have been oriented such that high-to-low concentration gradients of BSA (vehicle control) or Wnt5a are highest at the top of the images. (Top panel) Control growth cones in BSA continue straight trajectories. (Middle panels) Three different growth cones show marked repulsive turning in Wnt5a gradients. (Bottom panel) Transfection with CaMKIIN abolishes Wnt5a induced repulsion. Scale bars, 10 μm. (B) A graph of fluorescence intensity (*Z* axis) of a gradient of 40 kDa Texas Red dextran at different positions in the bridge region of the Dunn chamber. A high-to-low gradient (along the *X* axis) is formed from the edge of the bridge region facing the outer chamber containing Texas Red dextran (0 μm) to the edge facing the inner chamber lacking Texas Red dextran. This gradient persists for at least 2 h (*Y* axis). (C) Rates of outgrowth of control- or CaMKIIN-transfected axons in Dunn chambers treated with gradients of BSA or Wnt5a. (D) Cumulative distribution graph of turning angles of control- or CaMKIIN-transfected axons in Dunn chambers treated with gradients of BSA or Wnt5a. **p < 0.01, Wilcoxon signed rank test. (E) Graph of turning angles of control- or CaMKIIN-transfected axons in Dunn chambers treated with gradients of BSA or Wnt5a. **p < 0.01, ANOVA on Ranks with Dunn's posttest.

Since guidance errors in the callosum by Ryk knockout were caused by interfering with Wnt5a induced cortical axon repulsion (Keeble et al.,^2006^), we asked whether CaMKII is also required for cortical axon repulsion. To address this question we used a Dunn chamber turning assay (Yam et al.,^2009^) in which cortical neurons were exposed to a Wnt5a gradient (Supporting Information Fig. S3) and their growth cone turning angles measured over 2 h. As shown in Figure 6(B), measurement of the Wnt5a gradient in the Dunn chamber, as measured with a fluorescent dextran conjugate similar in molecular weight to Wnt5a, showed that a high to low Wnt5a gradient was established in the bridge region of the chamber that persisted for the 2-h duration of the experiments. As we found previously in a pipette turning assay (Li et al.,^2009^), growth cones of neurons in the bridge region of the Dunn chamber consistently turned away from Wnt5a gradients and increased their growth rates by 50% [Figs. 6(C–E) and S4]. In contrast when cortical neurons were transfected with CaMKIIN they failed to increase their rates of axon growth [Fig. 6(C)]. Importantly inhibition of CaMKII prevented axons from repulsive turning in response to Wnt5a and these axons continued extending in their original trajectories [Fig. 6(D,E)]. These results suggest that, as with inhibition of Ryk receptors (Li et al.,^2009^), reducing CaMKII activity slows axon outgrowth and prevents Wnt5a growth cone repulsion.

## DISCUSSION

Taken together these results show that in a cortical slice model of the developing corpus callosum Wnt/calcium signaling pathways, that we previously identified in dissociated cortical cultures (Li et al.,^2009^), are essential for regulating callosal axon growth and guidance. First we show that rates of callosal axon outgrowth are almost 50% higher on the contralateral side of the callosum. Second we find that higher frequencies of calcium transients in postcrossing growth cones are strongly correlated with higher rates of outgrowth in contrast to precrossing growth cones. Third we show that blocking IP3 receptors with 2-APB slows the rate of postcrossing axon growth rates but does not affect axon guidance. In contrast blocking TRP channels not only reduces axon growth rates but causes misrouting of postcrossing callosal axons. Downstream of calcium, we found that CaMKII is essential for normal axon growth and guidance, demonstrating the importance of calcium signaling for development of the corpus callosum. Finally, we discovered that knocking down Ryk expression reduces postcrossing axon outgrowth and induces aberrant trajectories. Importantly we show that these defects in axons treated with Ryk siRNA correspond with reduced calcium activity. These results suggest a direct link between calcium regulation of callosal axon growth and guidance and Wnt/Ryk signaling.

Although calcium transients in growth cones of dissociated neurons have been extensively documented in regulating axon outgrowth and guidance (Henley and Poo,^2004^; Gomez and Zheng,^2006^; Wen and Zheng,^2006^), the role of axonal calcium transients has been little studied *in vivo*. A previous live cell imaging study of calcium transients *in vivo* in the developing Xenopus spinal cord demonstrated that rates of axon outgrowth are inversely related to frequencies of growth cone calcium transients (Gomez and Spitzer,^1999^). Here we show that callosal growth cones express repetitive calcium transients as they navigate across the callosum. In contrast to results in the Xenopus spinal cord, higher levels of calcium activity are correlated with faster rates of outgrowth. One possibility to account for these differences is that in callosal growth cones calcium transients were brief, lasting ∼1 s, whereas in Xenopus spinal growth cones calcium transients were long lasting, averaging almost 1 min (Gomez and Spitzer,^1999^; Lautermilch and Spitzer,^2000^). Thus calcium transients in Xenopus that slow axon outgrowth could represent a different kind of calcium activity, consistent with the finding that rates of axon outgrowth in dissociated spinal neuronal cultures were insensitive to inhibitors of CaMKII (Zheng et al.,^1994^; Lautermilch and Spitzer,^2000^). In dissociated cortical cultures calcium activity in growing axons was similar in frequency and duration to callosal growth cones extending in slices (Hutchins and Kalil,^2008^). Some callosal growth cones exhibit calcium activity localized to the growth cone or even small regions of the growth cone, raising the possibility that asymmetries in levels of calcium could play a role in growth cone steering *in vivo* as they do in isolated growth cones (Henley and Poo,^2004^). Thus the present study is the first to demonstrate the importance of repetitive calcium transients for axon outgrowth and guidance in a developing mammalian CNS pathway.

Previous studies have shown the importance of the source of calcium activity for effects on axon growth and guidance (Ooashi et al.,^2005^; Jacques-Fricke et al.,^2006^). For example, transients resulting from calcium entry through L-type channels was found to inhibit axon outgrowth in dissociated cortical cultures (Tang et al.,^2003^; Hutchins and Kalil,^2008^). In contrast calcium release from stores through IP3 receptors promotes axon outgrowth (Takei et al.,^1998^; Jacques-Fricke et al.,^2006^; Li et al.,^2009^). In the present study blocking IP3 receptors reduced rates of axon outgrowth by about 50% on the postcrossing side of the callosum, showing for the first time that axons growing in developing mammalian pathways use similar calcium signaling mechanisms to regulate their growth rates. Recent *in vitro* studies of axon guidance in response to application of netrin-1 or BDNF have shown the importance of calcium entry via TRP channels to induce attractive or repulsive growth cone turning (Li et al.,^2005^; Shim et al.,^2005^; Wang and Poo,^2005^). Similarly we found that in dissociated cortical cultures repulsive turning of cortical growth cones in Wnt5a gradients were inhibited when TRP channels were blocked (Li et al.,^2009^) although this also reduced rates of axon outgrowth. This result is consistent with the recent finding that pharmacologically blocking TRP channels or knocking down TRPC5 reduces rates of hippocampal axon outgrowth (Davare et al.,^2009^). Here we find that application of TRP channel blockers to cortical slices blocks calcium transients and reduces rates of callosal axon outgrowth but also causes severe misrouting of callosal axons. This demonstrates the requirement of TRP channels for axon guidance in the mammalian CNS.

Although these results show the importance of calcium signaling in regulating callosal growth and guidance, calcium activity could be evoked by multiple guidance cues. For example, sources of netrins, semaphorins, and Slit2 surround the corpus callosum and their role in callosal axon guidance across the midline has been well characterized (Serafini et al.,^1996^; Shu and Richards,^2001^; Shu et al.,^2003^; Lindwall et al.,^2007^; Niquille et al.,^2009^; Piper et al.,^2009^). However, our finding that inhibiting calcium signaling only affected growth and guidance of axons after but not before the callosal midline suggested that these effects were due to axonal responses only after they had crossed the midline. This points to the possible involvement of Wnt5a signaling, because, cortical axons do not respond to Wnt5a until the age at which they cross the midline (Keeble et al.,^2006^). Although Slit2 affects both pre- and postcrossing callosal axons (Shu and Richards,^2001^; Shu et al.,^2003^), our results showed that interfering with calcium/CaMKII affected only postcrossing axon outgrowth. This result points to a mechanism that is specific to the contralateral hemisphere such as Wnt5a signaling. This interpretation is supported by our similar results on postcrossing axon outgrowth after knocking down the Ryk receptor. We recently showed that Wnt/Ryk signaling evokes repetitive calcium transients in cortical axons in dissociated cultures (Li et al.,^2009^). Although Wnt5a is thought to be the ligand for Ryk in the developing corpus callosum (Keeble et al.,^2006^), it is possible that other Wnt proteins contribute to Ryk-dependent outgrowth and guidance in the contralateral callosum. We demonstrate here that knocking down the Ryk receptor reduces rates of callosal axon outgrowth and that, in axons lacking Ryk receptors, growth, and guidance errors coincide with reduced calcium activity. Since Wnts are currently the only known ligands for the Ryk receptor (Fradkin et al.,^2010^), this remarkable result provides compelling evidence that calcium regulation of postcrossing axon growth and guidance in the callosum is specifically evoked by Wnt signaling.

Although it is possible that signaling pathways other than Wnt/Ryk activate downstream calcium/CaMKII signaling in callosal axons, the selective effects of inhibiting CaMKII on postcrossing axon outgrowth suggest that Ryk is a primary activator of the CaMKII signaling cascade. Our work *in vitro* indicates that CaMKII is specifically phosphorylated by Wnt5a signaling (our unpublished observations). Frequencies of calcium transients were 75% lower in axons on the precrossing side of the callosum, where Ryk is not active (Keeble et al.,^2006^). This result suggests that guidance cues affecting callosal axons in that region do not promote outgrowth through frequency dependent calcium signaling, which is consistent with our finding that inhibiting CaMKII had no effect on the growth of precrossing axons. Furthermore, our results which show that CaMKII promotes axonal growth selectively in regions of high-frequency calcium activity suggests an instructive rather than permissive role for CaMKII in regulating growth cone advance. This interpretation is supported by our finding that experimentally reducing frequencies of calcium transients also reduces rates of axon outgrowth to levels seen in precrossing axons with naturally low calcium activity. The lack of any additive effects when calcium transients are pharmacologically suppressed in axons expressing the CaMKII inhibitor CaMKIIN (Supporting Information Fig. S5) indicates that CaMKII does not have any calcium frequency-independent effects in callosal axons, further demonstrating an instructive role for CaMKII in callosal axon outgrowth.

**Figure 7:**
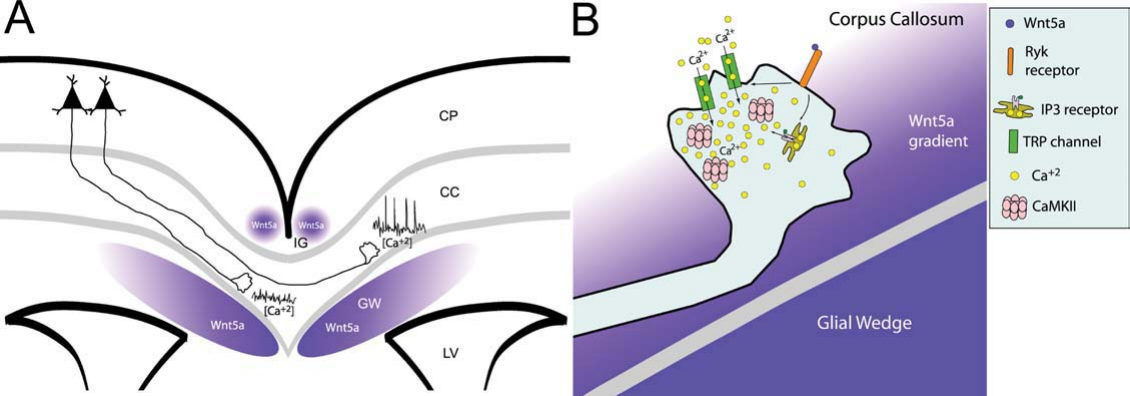
Wnt/calcium signaling guiding axon growth through the corpus callosum. (A) A schematic of a cortical slice showing cortical axons extending through the corpus callosum (CC) around which Wnt5a is expressed in the induseum griesium (IG) and glial wedge (GW). Axons in the contralateral callosum have high frequencies of calcium transients caused by Wnt5a signaling which accelerate rates of axon outgrowth. In contrast, ipsilateral axons, which are not yet responsive to Wnt5a, have little calcium activity and therefore grow more slowly. LV, lateral ventricle; CP, cortical plate. (B) A schematic of intracellular signaling in the post-crossing axon from (A). A Wnt5a gradient from the glial wedge activates Ryk receptors to open IP3 receptors and TRP channels, causing release of calcium from intracellular stores and calcium influx through the plasma membrane. These transients activate CaMKII to accelerate axon extension and repel growth cones away from the glial wedge toward the contralateral cortical plate.

Taken together, our results from dissociated cortical cultures (Li et al.,^2009^) and the present findings in cortical slices support a repulsive guidance function for Wnt5a on cortical axons (see Fig. 7) in agreement with previous studies (Liu et al.,^2005^; Keeble et al.,^2006^; Zou and Lyuksyutova,^2007^). However, calcium signaling mechanisms underlying growth cone turning in response to guidance cues remain poorly understood. One recent study, on the basis of asymmetric membrane trafficking in growth cones with calcium asymmetries, suggested that attraction and repulsion are not simply opposite polarities of the same mechanism but distinct mechanisms (Tojima et al.,^2007^). Axon growth and turning behaviors in response to attractive cues such as BDNF (Song et al.,^1997^; Li etal.,^2005^; Hutchins and Li,^2009^) and netrin-1 (Hong et al.,^2000^; Henley and Poo,^2004^; Wang and Poo,^2005^) or turning away from repulsive cues such as myelin-associated glycoprotein (MAG), (Henley et al.,^2004^) involve Ca^2**+**^ gradients in growth cones with the elevated side facing toward the source of the guidance cue (Zheng et al.,^1994^; Henley and Poo,^2004^; Wen et al.,^2004^; Jin et al.,^2005^; Gomez and Zheng,^2006^). One model of calcium signaling in growth cone turning proposed that the amplitude of calcium gradients was higher in attractive growth cone turning but lower in repulsion (Wen et al.,^2004^). These different calcium gradients are detected by different calcium sensors such that high amplitude calcium signals in attraction are detected by CaMKII and low amplitude signals in repulsion are detected by calcineurin. Thus our finding that CaMKII is involved in growth cone repulsion is surprising given that a role for CaMKII has only been described for chemoattraction (Wen et al.,^2004^; Wen and Zheng,^2006^). Furthermore, the finding that CaMKII is required for axon guidance in the callosum emphasizes the importance of these calcium-dependent guidance behaviors *in vivo*. A previous study of calcium signaling pathways activating CaMKK and CaMKI reported no axon guidance or extension defects during midline crossing, but rather showed reduced axon branching into cortical target regions (Ageta-Ishihara et al.,^2009^). This result suggests that distinct calcium/calmodulin kinases may be activated during different stages of callosal development. Taken together, these findings indicate that Wnt5a-evoked calcium/CaMKII signaling instructs specific growth and guidance behaviors in the corpus callosum rather than reflecting a more general requirement for calcium signaling during development.
