# Supplementary figures and images for: Wnt/Calcium Signaling Mediates Axon Growth and Guidance in the Developing Corpus Callosum
Source: Dev Neurobiol. 2010 Oct 8;71(4):269–83. doi: 10.1002/dneu.20846 (PMC3099647; doi:10.1002/dneu.20846)

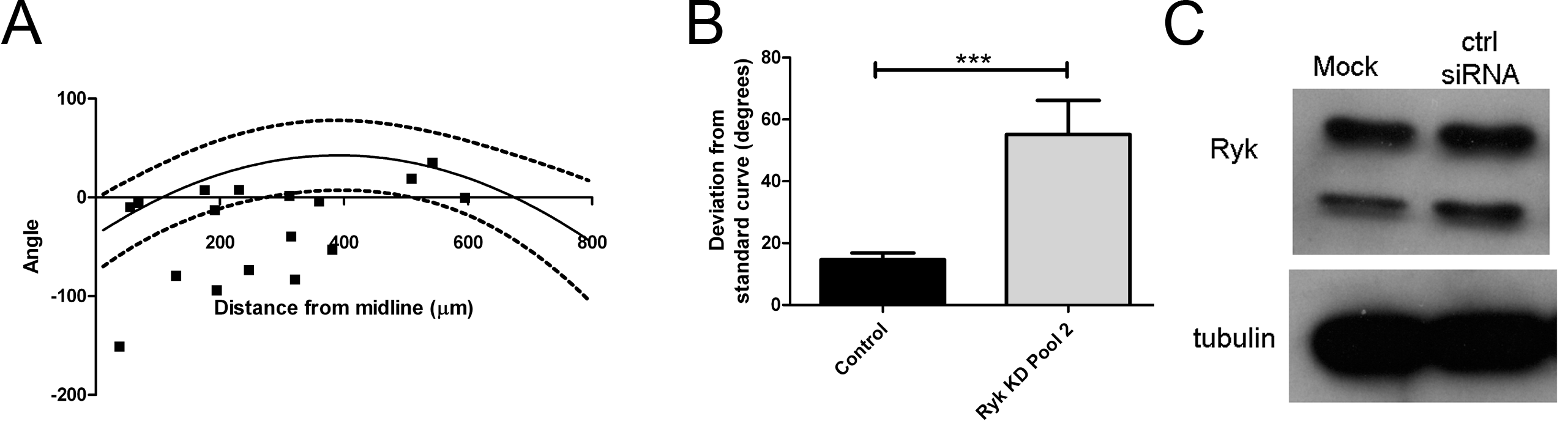

Supplement: Supplementary file 1 [file dneu0071-0269-SD1.tif]

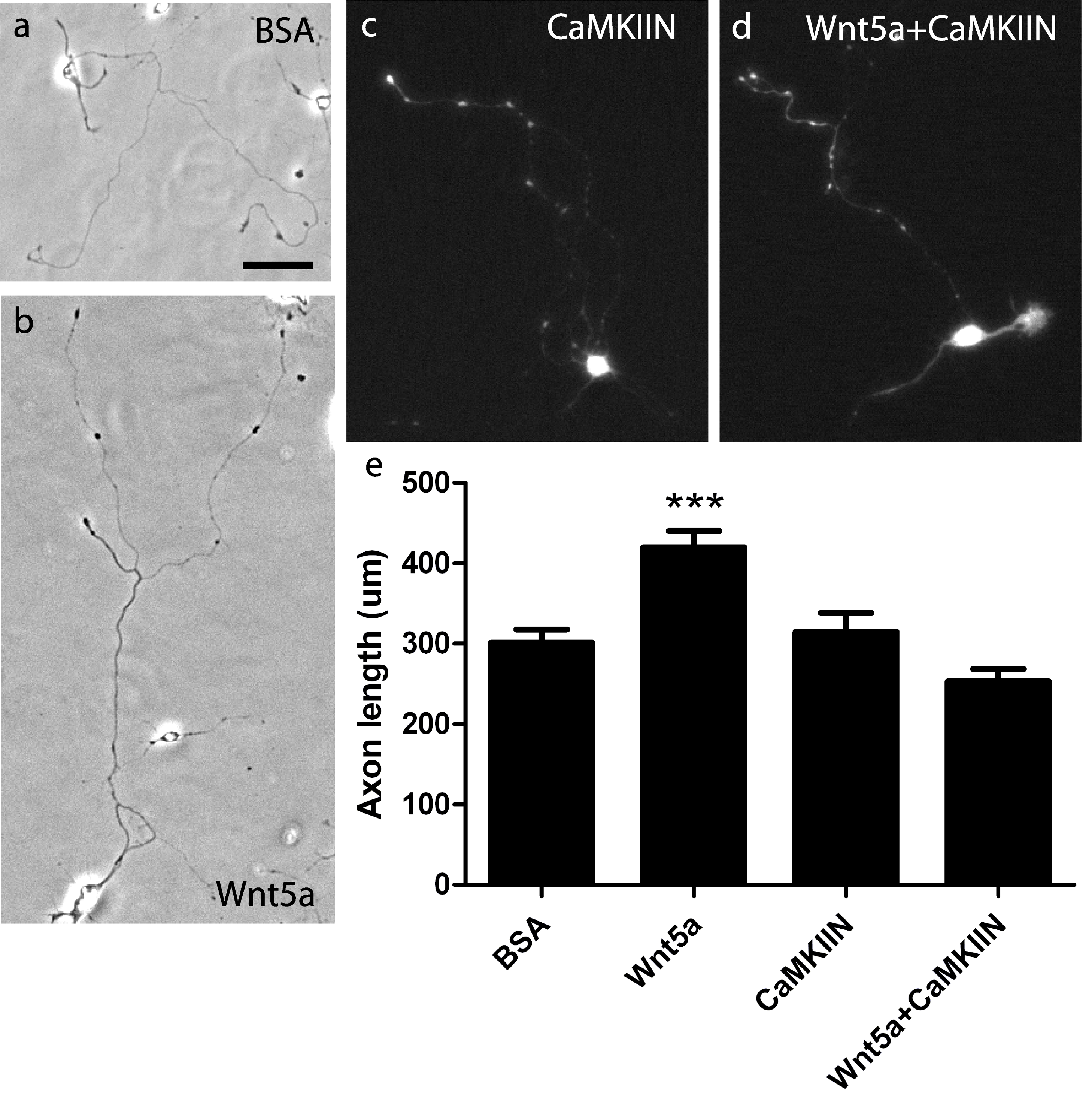

Supplement: Supplementary file 2 [file dneu0071-0269-SD2.tif]

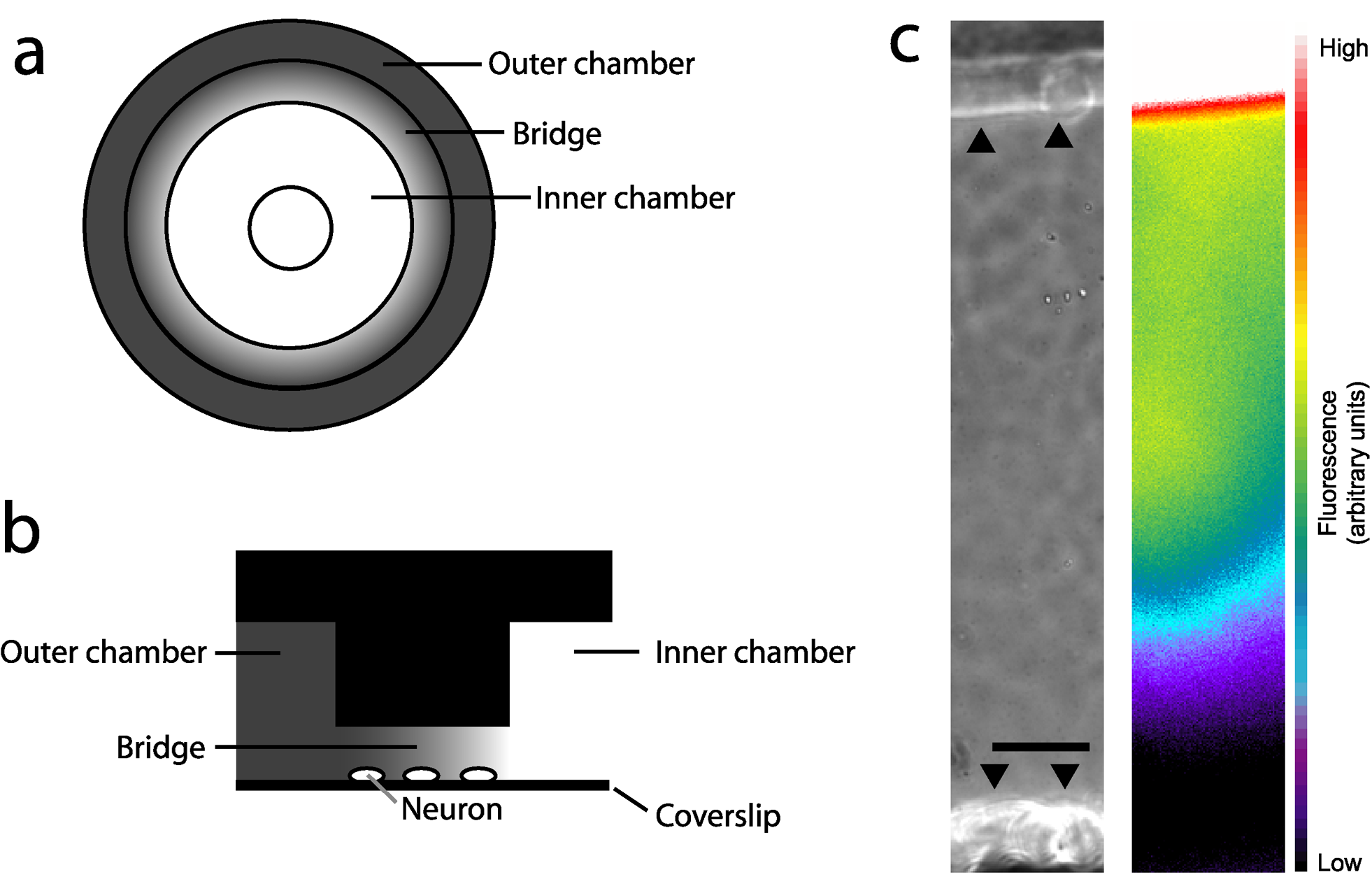

Supplement: Supplementary file 3 [file dneu0071-0269-SD3.tif]

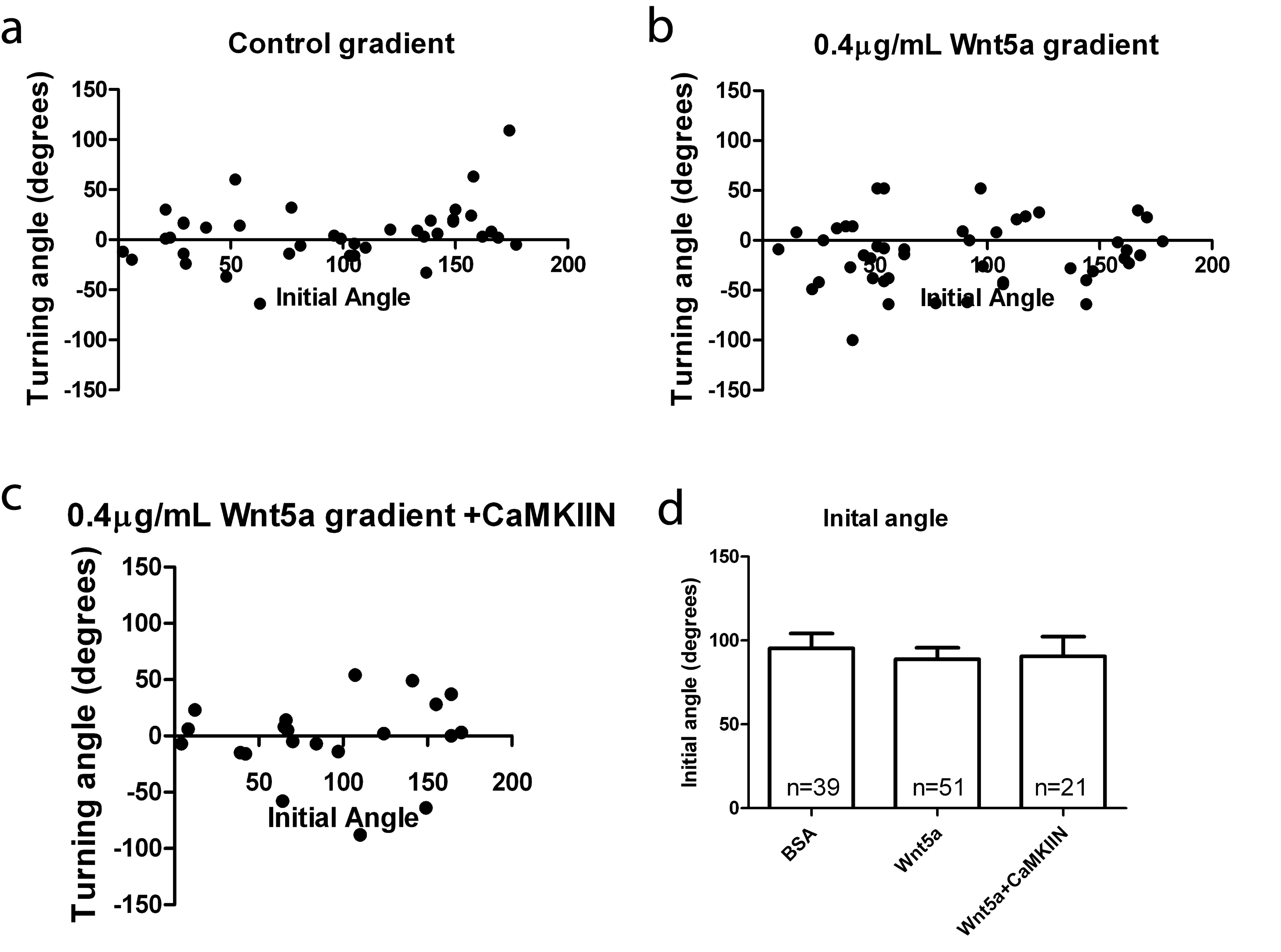

Supplement: Supplementary file 4 [file dneu0071-0269-SD4.tif]

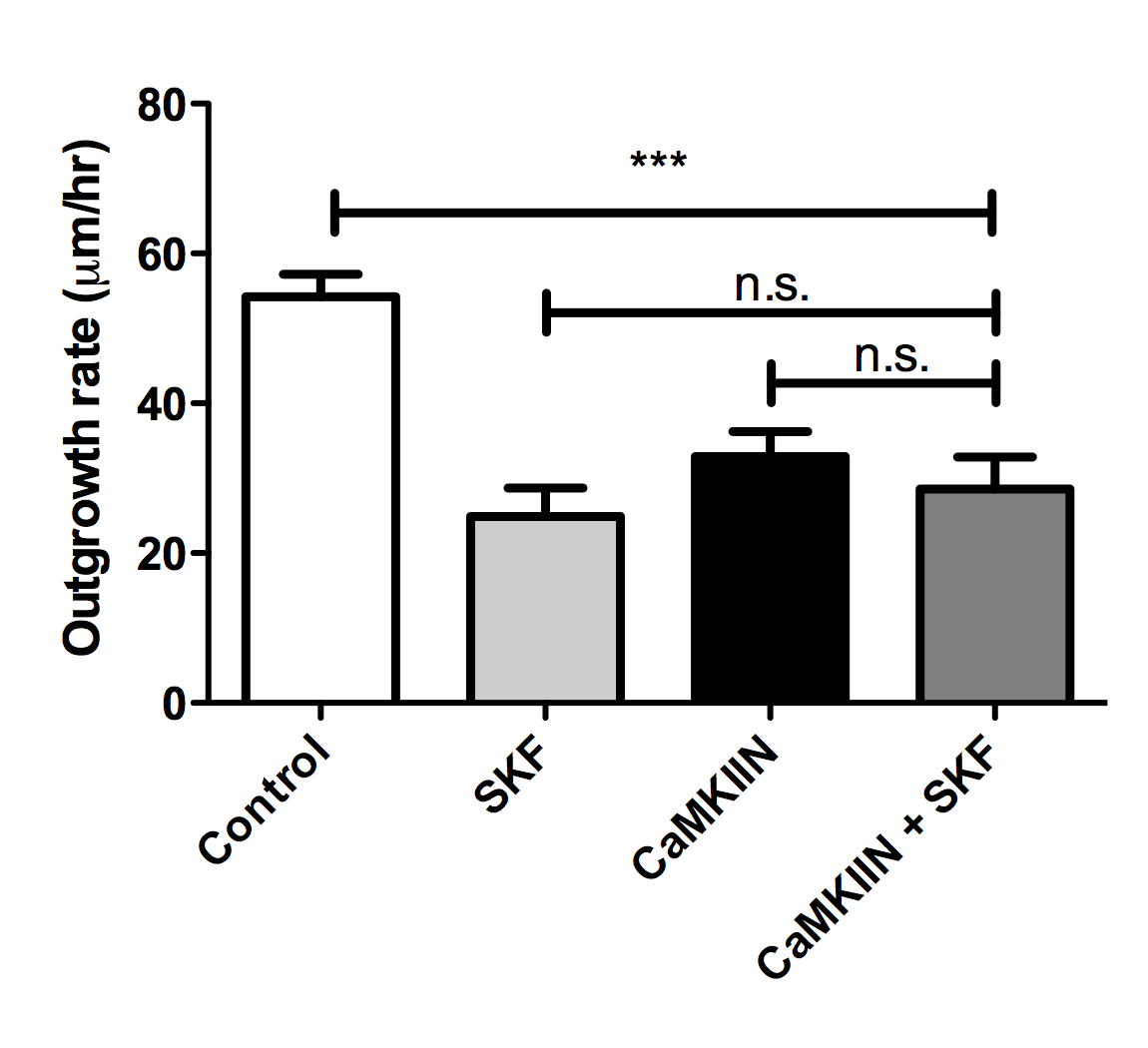

Supplement: Supplementary file 5 [file dneu0071-0269-SD5.tif]
